# Pancreatic undifferentiated carcinoma with osteoclast-like giant cells curatively resected after pembrolizumab therapy for lung metastases: a case report

**DOI:** 10.1186/s12876-020-01362-4

**Published:** 2020-07-11

**Authors:** Miku Obayashi, Yasushi Shibasaki, Toru Koakutsu, Yoshiro Hayashi, Tsuyoshi Shoji, Kazuhisa Hirayama, Masanori Yamazaki, Yasuhiro Takayanagi, Hiroshi Shibata, Masato Nakamura, Hirotoshi Maruo

**Affiliations:** 1 Department of Surgery, Shizuoka City Shimizu Hospital, 1231 Miyakami, Shimizu-ku, Shizuoka, 424-8636 Japan; 2 Department of Gastroenterology, Shizuoka City Shimizu Hospital, 1231 Miyakami, Shimizu-ku, Shizuoka, 424-8636 Japan; 3 Department of Respiratory Medicine, Shizuoka City Shimizu Hospital, 1231 Miyakami, Shimizu-ku, Shizuoka, 424-8636 Japan; 4 Department of Pathology, Shizuoka City Shimizu Hospital, 1231 Miyakami, Shimizu-ku, Shizuoka, 424-8636 Japan

**Keywords:** Undifferentiated carcinoma with osteoclast-like giant cells, UCOGC, Pancreatic ductal adenocarcinoma, Lung metastasis, Pembrolizumab, PD-1, PD-L1, Mismatch repair, Microsatellite instability, Case report

## Abstract

**Background:**

Therapy targeting programmed death-1 or programmed death-1 ligand-1 (PD-1/PD-L1) has been developed for various solid malignant tumors, such as melanoma and non-small-cell lung cancer (NSCLC), but this approach has little effect in the treatment of pancreatic cancer. Pancreatic undifferentiated carcinoma with osteoclast-like giant cells (UCOGC) is a rare pancreatic malignancy having unique morphology and is considered a variant of pancreatic ductal adenocarcinoma (PDAC). Although UCOGC has been reported to have better prognosis than conventional PDAC, the optimal treatment for UCOGC with distant metastases has not been determined.

**Case presentation:**

A 66-year-old man was initially diagnosed with NSCLC with multiple intrapulmonary metastases and abdominal lymph node metastasis in the tail of the pancreas, and bronchial biopsy and diagnostic imaging were performed. Pathologic examination of the lung showed poorly differentiated adenocarcinoma cells expressing epithelial marker and PD-L1. Therefore, pembrolizumab monotherapy for NSCLC was given. The pulmonary lesions shrank markedly and were in complete remission after 8 months of anti-PD-1 therapy, though no therapeutic effect was observed in the pancreatic site. Distal pancreatectomy was then performed, and histopathological examination showed that the tumor was UCOGC originating from the pancreas. The histologic findings of the resected specimen mimicked those of the lung biopsy specimen, leading to the final assessment that the lung tumors were metastatic foci that migrated from the UCOGC, and only the metastatic lesions benefited from pembrolizumab therapy.

**Conclusion:**

Immune checkpoint inhibitors have limited therapeutic effects on primary lesions of pancreatic cancer, but they may exert antitumor effects on pulmonary metastases of UCOGC.

## Background

Pancreatic cancer (PC) is a highly aggressive malignancy with a 5-year overall survival rate of 9% [1], and its incidence is increasing. Pancreatic undifferentiated carcinoma with osteoclast-like giant cells (UCOGC) is an extremely rare tumor, accounting for 1.4% of invasive PCs [2], and its prognosis has been reported to be better than that of typical pancreatic ductal adenocarcinoma (PDAC) in surgically resected cases [2, 3]. But the optimal treatment for UCOGC with distant metastases is unknown. Because of the difficulties in early diagnosis, curative surgery, and chemo-resistance leading to a poor prognosis, it is imperative to establish an effective treatment approach for PC. Recently, immune checkpoint inhibitors, such as anti-programmed death-1 or programmed death-1 ligand-1 (PD-1/PD-L1) antibody, have been developed for various solid carcinomas and have shown broad efficacy [4, 5], but they have a poor effect in the treatment of PC, as seen with PD-1/PD-L1 blockade monotherapy [6, 7]. Previous studies suggested that the tumor microenvironment (TME) plays key roles in the immunotherapy failure mechanism, with abundant stromal desmoplasia and/or tumor-infiltrating lymphocytes (TILs) [8, 9]. In addition, DNA mismatch repair (MMR) deficiency is an important factor for immune checkpoint inhibitor sensitive mechanism in solid tumors [10, 11]. Pembrolizumab, a humanized monoclonal antibody against PD-1, has been reported to have strong antitumor activity in advanced non-small-cell lung cancer (NSCLC) [5, 12], although it has not shown sufficient therapeutic effects in PC. A case of UCOGC that was curatively resected following pembrolizumab monotherapy that was highly effective for metastatic lung cancer is presented.

## Case presentation

**Fig. 1:**
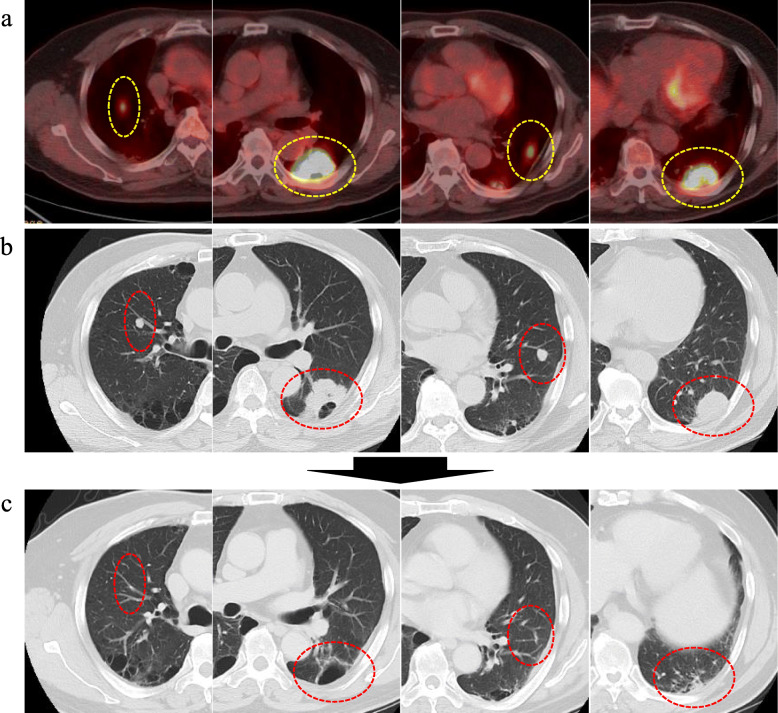
Preoperative images of the lung tumor. **a** Positron emission tomography (PET) before pembrolizumab therapy shows multiple tumors in bilateral lobes of the lungs (dotted circles). Marked fluorodeoxyglucose (FDG) uptake is observed in each panel (standardized uptake value (SUV) max 14.7 in the second left panel, for example). **b** Chest computed tomography (CT) before pembrolizumab therapy shows 4 nodules corresponding to each point of PET-CT (dotted circles). **c** CT imaging after pembrolizumab therapy. Almost complete remission is seen (dotted circles)

**Fig. 2:**
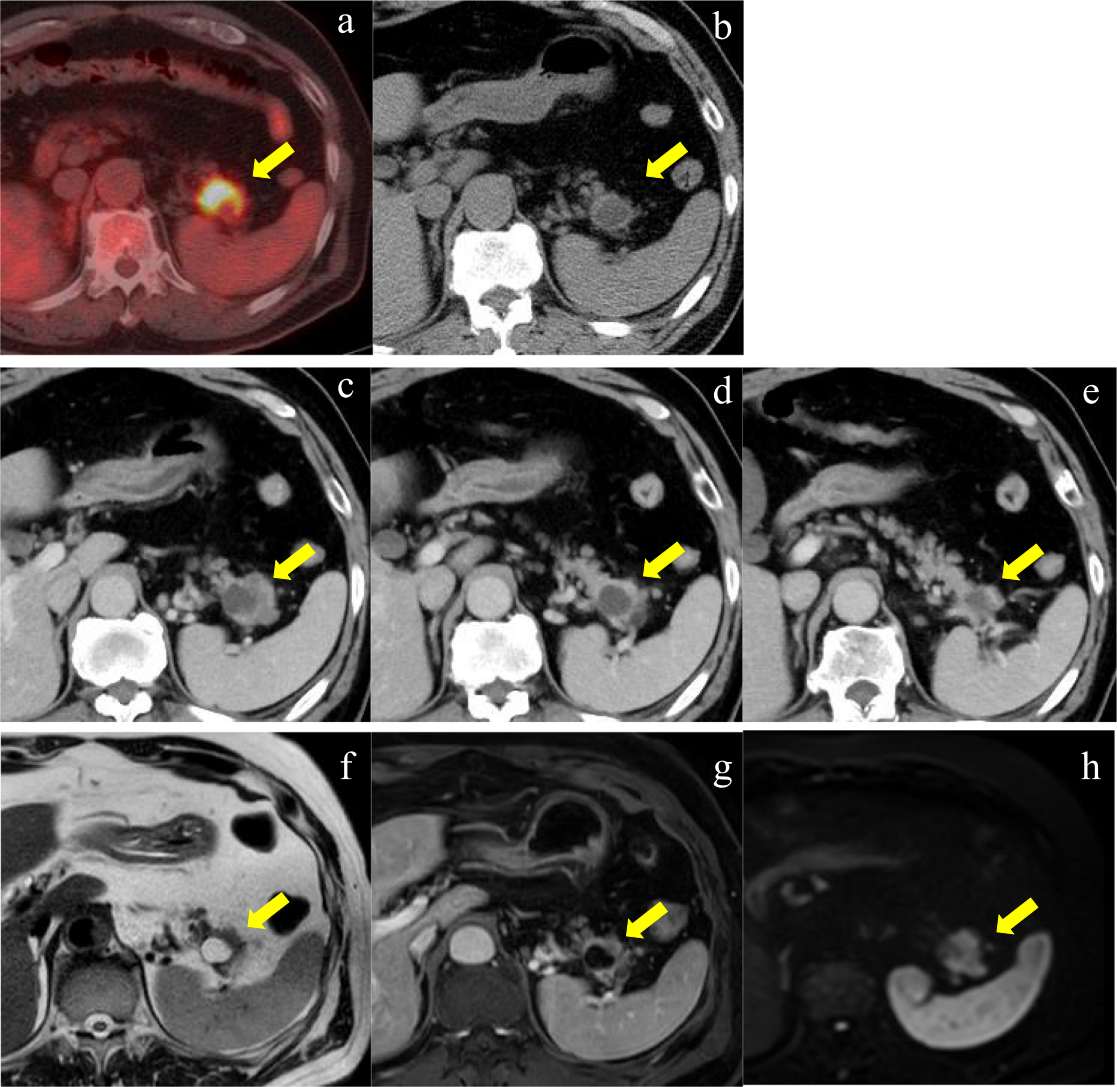
Preoperative images of the pancreatic tumor. **a** PET-CT before pembrolizumab therapy shows a solitary mass in the splenic hilum (arrow; SUVmax: 12.8). **b** Abdominal CT before pembrolizumab therapy shows a low-density mass in the splenic hilum, about 20 mm in diameter (arrow). **c**-**e** A series of contrast-enhanced CT scans after pembrolizumab therapy. The mass shows slight expansion up to 25 mm in size. This tumor is located in the tail of the pancreas, including a cystic lesion surrounded by a solid component that is irregularly enhanced in the delayed phase (arrows). **f**-**h** Magnetic resonance imaging (MRI) after pembrolizumab therapy. The tumor (arrows) is composed of a high-intensity moiety on the T2-weighted image (f), and surrounding parenchyma is enhanced by contrast medium in the delayed phase (g) and shows high intensity on the diffusion-weighted image (h)

**Fig. 3:**
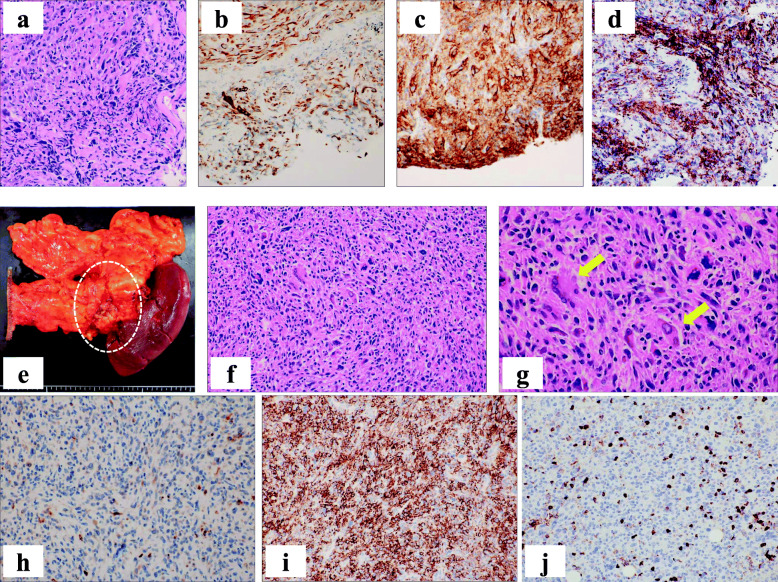
Pathological examination of the lung and pancreas. **a**-**d** Transbronchial lung biopsies: The cancerous cells show pleomorphic atypical cells containing hyperchromatic nuclei in the pulmonary lesion, forming abortive glands and solid nests with fibrous stroma (**a**, HE, original magnification × 200). Immunohistochemical stains show cytokeratin (CK)-7-positive (**b**, original magnification × 200) and programmed death ligand-1 (PD-L1) cancer cells (**c**, original magnification × 200). Leukocyte common antigen (LCA) is also positive in stromal cells (**d**, original magnification × 200). **e**-**j** Pancreatic lesion: The hard tumor is located on the ventral side of the tail of the pancreas with anterior invasion to the stomach (dotted circle in **e**). Sarcomatoid appearance with spindle-shaped cells and pleomorphic multinucleated cells (**f**, HE, original magnification × 200). Osteoclast-like giant cells (OGCs) are also seen (arrows) in the tumor (**g**, HE, original magnification × 400). Immunohistochemistry: CK-7 (**h**) and PD-L1 (**i**) are also positive. Small amount of lymphocytic infiltration around the cancer cells was observed in LCA staining (**j**, original magnification × 200)

A 66-year-old man visited our hospital because of abnormal lung shadows found on screening chest X-ray examination. Positron emission tomography (PET) and computed tomography (CT) showed multiple nodules in bilateral lung lobes (Fig. 1a and b) and a solitary mass in the splenic hilum (Fig. 2a and b). Lung biopsy from the left middle lobe showed poorly differentiated adenocarcinoma (Fig. 3a), the cells of which were immunohistochemically positive for cytokeratin (CK)- Wide Spectrum Screening (WSS) and CK-7 (Fig. 3b). Based on these findings, this patient was diagnosed as having primary NSCLC with multiple metastases to bilateral lobes and abdominal lymph node, since the mass in the tail of the pancreas was initially considered to be splenic hilum lymph node metastasis. Mutation of epidermal growth factor receptor (EGFR) and the expression of anaplastic lymphoma kinase (ALK) were negative. The PD-L1 immunohistochemistry (IHC) was then performed using anti-PD-L1 antibody (Dako, Carpinteria, CA, clones: 22c3, pharmDx assay; Dilution 1:50). Sections (4-μm thick) were prepared from formalin-fixed and paraffin-embedded (FFPE) tissues, and staining for 22c3 was performed on the Dako Link-48 autostainer system. PD-L1 expression was positive in nearly all cancer cells (Fig. 3c). Lymphocytic infiltration was abundantly observed in cancer tissue by immunohistochemical analysis using leukocyte common antigen (LCA) (Fig. 3d). Pembrolizumab monotherapy was then given. After 8 months, almost complete remission was observed in the lung tumors (Fig. 1c), whereas the size of the pancreatic mass did not decrease (Fig. 2c-e) on CT examination. This pathology raised the possibility that this pancreatic tumor might be a primary pancreatic malignancy independent of NSCLC. Contrast-enhanced CT showed a hypodense mass with peripheral irregular enhancement in the tail of the pancreas (Fig. 2c-e). Magnetic resonance imaging (MRI) also showed a high-intensity mass on T2 and diffusion-weighted imaging, suggesting high cellularity with a cystic component (Fig. 2f-h). From these findings, this tumor was diagnosed as a primary pancreatic neoplasm unaffected by pembrolizumab, such as PDAC or a neuroendocrine tumor. After appropriate informed consent was obtained from the patient, distal pancreatectomy with splenectomy was performed about 1 year after initial diagnosis. The resected specimen showed a solid tumor, 42 mm in size, on the ventral side of the tail of the pancreas, with slight invasion into the stomach wall (Fig. 3e). Histopathological examination showed pleomorphic atypical cells, including spindle-shaped cancer cells and osteoclast-like giant cells (OGCs) (Fig. 3f and g), which infiltrated to surrounding parenchyma and the splenic vein. Regional lymph nodes showed metastatic foci. Immunohistochemically, the tumor cells were positive for CK-WSS and CK-7 (Fig. 3h), and PD-L1 was also highly expressed in the tumor cells (Fig. 3i). There was small number of lymphocytes which were positive for LCA around the tumor cells, indicating scarce lymphocytic infiltration (Fig. 3j). Based on these findings, this malignancy was diagnosed as a UCOGC that arose from the pancreas. Furthermore, since these pathological characteristics were extremely similar to those of the pulmonary cancer, the diagnosis of the lung tumor as NSCLC was reconsidered. Finally, it was determined that UCOGC was the primary tumor that originated from the pancreas, and the pulmonary lesions were metastatic foci from the PC, and that pembrolizumab had little effect on the primary, but, curiously, was dramatically effective for the metastases. Microsatellite instability (MSI) is a biomarker for immunotherapy, representing the DNA mismatch repair (MMR) deficiency which predicts the favorable response of pembrolizumab to some solid malignant tumors [10, 11]. In the present case, MSI was also investigated by polymerase chain reaction (PCR)- based testing which evaluates the five poly-A mononucleotide repeats (BAT-25, BAT-26, NR-21, NR-24, NR-27) [13], although repeat length alterations were not seen both in the metastatic and primary cancer specimen. This patient remains alive, and there are no signs of recurrence 6 months after the operation without anticancer therapy.

## Discussion and conclusions

UCOGC is a rare variant of PDAC having a unique morphology which is composed of non-neoplastic cells (OGCs) and neoplastic cells (spindle and pleomorphic cells) without a definitive direction of differentiation [14, 15]. UCOGC used to be considered as an aggressive type of PC, because of the confusion of other “undifferentiated” carcinoma derived from PDAC, mucinous neoplasms and anaplastic carcinoma [16, 17]. Recent studies investigated the clinical and pathological features of pure UCOGC and concluded that patients with UCOGC had a significantly better prognosis than that with conventional PDAC or UCOGC derived from PDAC [2, 3], although the genetic alterations in UCOGC are notably similar to ordinary PDAC [3]. OGCs are the hallmark of UCOGC and defined as cells having a histologic resemblance to giant cell tumors of the bone, containing pleomorphic, multinucleated and mononucleated giant cells [18], and an abundance of OGCs is reported to correlate with favorable prognosis of UCOGC [2]. Little is known about the mechanisms of such good prognoses regardless of tumor size, though UCOGCs have been reported to have few metastases to lymph nodes or perineural invasion [2]. In the present case, distinct ductal differentiation was not seen and a large number of OGCs was observed in tumor tissue, but both distant metastases and lymph node metastases were positive. Nevertheless, this patient has had a relatively good outcome after pembrolizumab therapy followed by curative resection.

Luchini et al. reported that UCOGCs showed relatively numerous tumor cells with PD-L1 expression, and PD-L1-positive UCOGCs had a worse prognosis than PD-L1-negative ones, and the mechanism of immune evasion is a feature of UCOGCs [19]. In the present case, PD-L1 was positively expressed in many tumor cells, but the outcome was acceptable. PD-1/PD-L1 expression is a biomarker that has been associated with an increased response rate to immunotherapy [11], albeit several problems about the methodology of IHC for PD-L1 have been discussed. Different antibodies and sets of assay condition for PD-L1 have been used for respective immune checkpoint inhibitors with contrasting results [20, 21], while the most used clone for PD-L1 is SP142 [11]. Luchini et al. used clone E1L3N for PD-L1 IHC in above mentioned study [19], whereas we used 22c3 which is approved to aid in selection of patients for pembrolizumab treatment based on PD-L1 expression in FFPE tumor samples [22]. It is reported that 22c3 was prone to show lower mean score of PD-L1 expression in IHC for NSCLC compared to E1L3N [21], therefore it is unlikely that our results of PD-L1 high expression in IHC was false positive. In fact, it is reasonable to assume that PD-1 antibody has normalized the cancer immune evasion system and has dramatically improved the prognosis of this patient.

Although there are no reports of anti-PD-1 therapy having sufficient effects in UCOGC with distant metastasis, a case in which pembrolizumab was very effective against the lung metastases of UCOGC was presented. PD-L1 expression in lung lesions was almost 100% in the present case, but it is known that the effect of anti-PD-1 antibody is not linked solely to the expression levels of PD-L1 in tumor tissues. It is well known that the TME, including TILs and tumor-associated macrophage (TAMs), is involved in the effect of immune checkpoint inhibitors [23]. The abundance of stromal desmoplasia also constitutes a principal characteristic of the PC TME [24]. In fact, PD-L1 was highly expressed in the tumor cells of PC, but pembrolizumab did not have a significant effect in the present case. However, the pancreatic tumor showed stable disease for several months, so it may have had some effect on the PC. Smyth et al. suggested that the TME should be categorized into four types based on the number of TILs and the PD-L1 expression level [8]. In the present case, PD-L1 expression was highly and equivalently confirmed in both lesions (Fig. 3c and i), whereas lymphocytic infiltration was stronger in the lung metastatic lesions than in the primary pancreatic lesion (Fig. 3d and j). Such variance in the TME might reflect the different effects of pembrolizumab at the two sites.

It has been reported that colorectal cancers with MMR deficiency are sensitive to immune checkpoint inhibitor [25]. Le et al. evaluated the sensitivity of MMR-deficient cancers to immune checkpoint inhibitor across 12 different tumor types including PCs, and the efficacy of pembrolizumab against MMR-deficient cancers was evaluated regardless of the cancer types [10]. In this study, complete responses were achieved in 21% of patients, verifying the hypothesis that large population of mutant neoantigens in MMR-deficient cancers make them sensitive to immune checkpoint blockage. However, MMR deficiency was observed less than 2% of pancreatic adenocarcinoma in advanced stage in this study [10], correspondingly in the present case with MSI negative. European Society for Medical Oncology (ESMO) recommends MMR IHC as a first method for MSI testing for immunotherapy [11], though MSI-PCR with five poly-A mononucleotides is also recommended as the current standard because of its high specificity and sensitivity [11, 13]. It is difficult to explain the present result that Pembrolizumab exerted sufficient efficacy against metastatic pulmonary lesions which was negative for MSI-PCR, exclusively by the MMR-deficient theory. Moreover, ESMO recommends novel next-generation sequencing (NGS) which permits a high-throughput sequencing of large number of microsatellites and genes with the determination of tumor mutational burden (TMB). TMB is also an emerging biomarker of sensitivity to immunotherapy and the abundance of TMB is known to correlate with the predictive upregulation of neoantigens [26], although we could not evaluate the TMB of UCOGC in this case. It was suggested that only 16% of cancers with high TMB were classified as MSI-high and the co-occurrence of these two phenotypes was highly dependent on the cancer type [27]. Le et al. also investigated the TMB in both primary and metastatic cancers in some cases, and showed the distinct difference of mutational genes and TMB between metastatic and primary cancers [10], indicating variable efficacy of immunotherapy depending on each organ. NGS-based MSI testing has the potential to become the method of choice for all tumor types, including rare cancers such as UCOGC.

Although MSI, TMB, tumor neoantigen and PD-1/PD-L1 expression are biomarkers that associated with response rate to immune checkpoint inhibitors, preferable response rates to immunotherapy has been described also in tumors without the expression of such biomarkers [28, 29]. Luchini et al. demonstrated the relationships among MSI, high TMB and PD-L1 expressions, and only 2.9% was positive for all these three parameters among the 4186 cancer patients [11]. Further studies are needed to find new biomarkers which can precisely predict the response to immunotherapy.

There are few reports of effective immune checkpoint inhibitors for PC, and many of them are investigations focusing on the effects on primary lesions. The validation of the effectiveness of anti-PD-1 antibody on distant metastases is a future issue to be considered. Because of genetical similarities, the establishment of the new treatment approach for UCOGC in advanced stage may be helpful to improve the prognosis of the patients with PDAC.

In conclusion, immune checkpoint inhibitors have limited therapeutic effects on primary lesions of PC, but they may exert antitumor effects on distant metastases of UCOGC.

## Data Availability

All data generated or analyzed during this study are included in this published article.

## Abbreviations

PD-1: Programmed death-1
PD-L1: Programmed death-1 ligand-1
NSCLC: Non-small-cell lung cancer
UCOGC: Undifferentiated carcinoma with osteoclast-like giant cells
PDAC: Pancreatic ductal adenocarcinoma
PC: Pancreatic cancer
TME: Tumor microenvironment
TIL: Tumor-infiltrating lymphocyte
MMR: Mismatch repair
PET: Positron emission tomography
CT: Computed tomography
CK: Cytokeratin
WSS: Wide spectrum screening
EGFR: Epidermal growth factor receptor
ALK: Anaplastic lymphoma kinase
IHC: Immunohistochemistry
FFPE: Formalin-fixed and paraffin-embedded
LCA: Leukocyte common antigen
MRI: Magnetic resonance imaging
OGC: Osteoclast-like giant cell
MSI: Microsatellite instability
PCR: Polymerase chain reaction
TAM: Tumor-associated macrophage
ESMO: European society for medical oncology
NGS: Next-generation sequencing
TMB: Tumor mutational burden
FDG: Fluorodeoxyglucose
SUV: Standardized uptake value
